# Contemporary pharmacological strategies for acute peripheral facial palsy: a narrative review with clinical decision considerations

**DOI:** 10.3389/fnins.2026.1840400

**Published:** 2026-06-23

**Authors:** Yupeng Guo, Xuanwei Dong

**Affiliations:** Department of Neurosurgery, Aviation General Hospital, Beijing, China

**Keywords:** acute peripheral facial palsy, antiviral therapy, Bell’s palsy, combination therapy, corticosteroids, facial nerve recovery, precision medicine, risk stratification

## Abstract

Acute peripheral facial palsy (APFP) sometimes known as Bell’s palsy is a common neurological condition and is marked by the acute onset of lower motor weakness on one side of the face. Whereas spontaneous recovery is widespread, there is a significant rate of incomplete recovery, synkinesis, or enduring functional and psychosocial disability of patients. The past decades witnessed the improvement of the diagnostic and therapeutic plans, particularly the pharmacological and adjunctive ones, due to the developments in pathophysiological and clinical trials and the creation of guidelines. This narrative review summarizes the existing evidence on the classification, diagnosis, and treatment of APFP, particularly corticosteroid disease treatment, antiviral medication, combination therapy, adjunct, and rehabilitative therapies, and the future of precision medicine. Randomised controlled trials and high-quality systematic reviews have shown evidence in support of the early initiation of systemic corticosteroids within 72 h of symptom onset as the foundation of treatment practice, improving the likelihood of achieving full facial nerve recovery and less morbidity in the long run. Conversely, antiviral monotherapy has not demonstrated significant clinical benefit with combination therapy with antivirals potentially presenting some benefit to older patients and with severe cases of paralysis. New data highlight the significance of risk stratification, electrophysiological testing, and focal rehabilitation to maximize the results and reduce the sequelae. The developments in artificial intelligence, the work on biomarkers and adaptive clinical trial designs will likely enable more personalized prognostication and treatment choice. In general, a shift towards precision risk-based approaches to the management of acute peripheral facial palsy is also being considered and emerging diagnostic strategies, promotion of the use of corticosteroids as early as possible and focused adjunctive operations that are tailored to a child are becoming the key to improving functional outcomes in the long term.

## Graphical abstract

**Figure:**
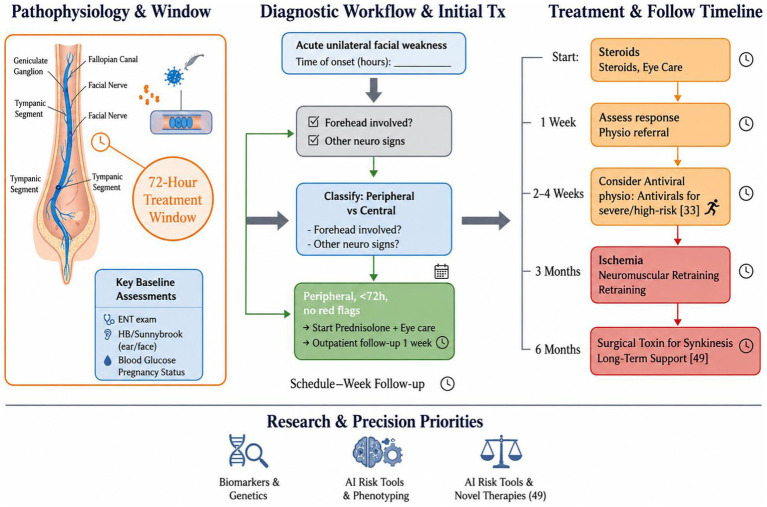
This diagram outlines a comprehensive, evidence-based management pathway for acute peripheral facial palsy (APFP/Bell’s palsy) that integrates early intervention, structured diagnostics, and precision medicine. The framework emphasizes the 72-h therapeutic window for corticosteroid initiation while incorporating systematic differentiation between peripheral and central etiologies. It presents a longitudinal care continuum—from acute-phase treatment through rehabilitation to long-term complication management—underpinned by emerging research directions in biomarkers, AI-driven risk stratification, and novel therapeutics, reflecting a paradigm shift toward personalized, risk-based clinical care.

## 1 Introduction

Acute peripheral facial palsy (APFP) is an acute neuropsychiatric disease that is acutely manifested by weakness or paralysis of one side of the face and is caused by a sudden dysfunction of the facial nerve (cranial nerve VII) (Madhok et al., 2016b; Gagyor et al., 2015b; Awantika and Prasad, 2022). Most of the cases are idiopathic and are referred to as Bell’s palsy, which is an approximation of 60–70 percentof unilateral facial paralysis, and 20–30 cases per 100,000 population per year across the globe (Dalrymple and Tiemstra, 2023; de Almeida et al., 2009a; Numthavaj et al., 2011a). Typically, in the clinical appearance, APFW results with rapidly progressive lower motor neuron facial weakness, which can have a spike in the severity of the condition in 48–72 h, and can be accompanied by retroauricular pain, dysgeusia, hyperacusia, or impaired lacrimation (Jalali et al., 2021). The pathophysiology of idiopathic APFP is still not fully clarified but there is growing evidence that a multifactorial mechanism is involved, that is inflammatory edema of the facial nerve in the narrow fallopian canal, resulting in ischemic damage and demyelination (Sullivan et al., 2009; Peitersen, 2002a; Tiemstra and Khatkhate, 2007). Viral reactivation especially that of Herpes simplex virus type 1 has also been suggested as a critical precipitant of the inflammatory cascade which can be biologically used to justify antiviral treatment in some cases (de Almeida et al., 2009a; Sullivan et al., 2009). Though there is spontaneous recovery in a reasonable percentage of patients, about 20–30% of the patients are left with incomplete recovery, persistent weakness of the face, synkinesis, or autonomic dysfunction that leads to serious functional deficiency and psychosocial burden (Peitersen, 2002a). The pharmacological intervention has thus become the key element of acute management that will help to enhance the results of recovery. The quality of randomized controlled clinical trials and systematic reviews is in agreement, showing that prompt corticosteroid treatment, especially at a time of 72 h after the initial symptoms appear, plays a major role in enhancing the full recovery of the facial nerve through micromanaging nerve swelling and edema (Tiemstra and Khatkhate, 2007; Hohman et al., 2025). As a result, corticosteroids are highly recommended as the initial treatment in the existing clinical guidelines (Dalrymple and Tiemstra, 2023; Tiemstra and Khatkhate, 2007). Conversely, the role of the antiviral agents in the therapy is still debatable. Randomized trials and meta-analyses have shown that antivirals are not associated with significant benefit over placebo and the regular use of antivirals in patients with Bell’s palsy is not justified (Gagyor et al., 2015b; Jalali et al., 2021). Although there is widespread evidence on combination therapy with corticosteroids and antivirals, there is evidence which indicates that the possible additive effect could only be seen in patients with severe facial paralysis or in patients with a presumed viral involvement (Peitersen, 2002a). This ambiguity has added to the heterogeneity in clinical practice and continued debate on the best pharmacological approaches.

**Figure 1:**
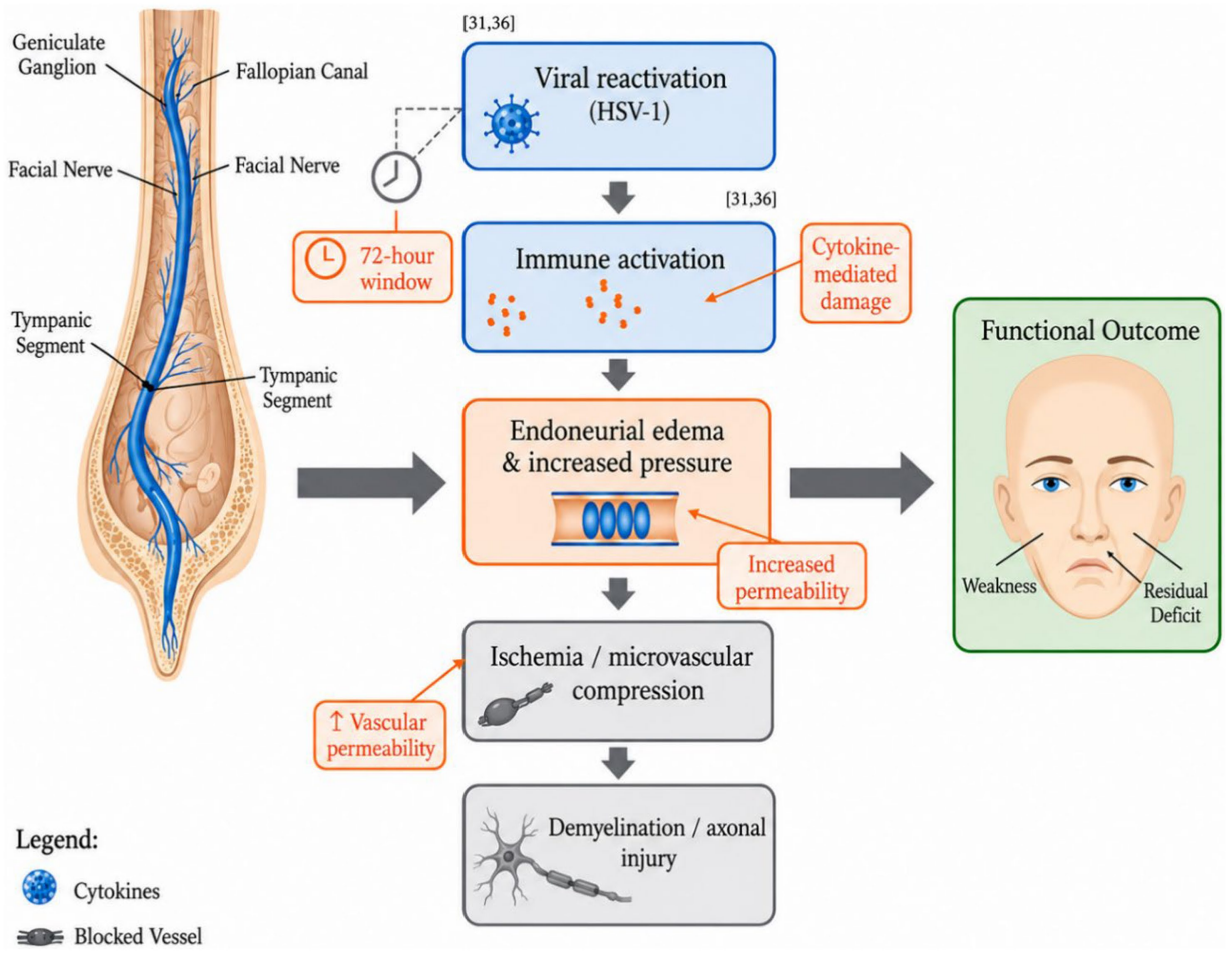
Proposed pathophysiological mechanisms in acute peripheral facial palsy. Schematic illustration depicting viral reactivation–induced inflammation, facial nerve edema within the fallopian canal, ischemic compression, and subsequent neural dysfunction leading to acute peripheral facial paralysis.

Since the evidence base is changing, there are still controversies, and there are emerging adjunctive therapies, a synthesis of the current pharmacological approaches should be presented in detail. The aim of this narrative review is to critically review available pharmacological interventions of APFP, synthesize evidence of randomized trials, systematic reviews, and clinical guidelines, and to give a decision consideration of clinical relevance and gaps that require further research in the future. Figure 1 illustrates the proposed pathophysiological cascade underlying APFP, highlighting the role of inflammatory edema and nerve compression within the fallopian canal that culminate in demyelination, axonal injury, and clinical facial weakness. The schematic emphasizes the time-sensitive window during which anti-inflammatory therapy is most likely to preserve neural function.

## 2 Classification and diagnosis

Proper classification and early diagnosis of APFP play a key role in proper management, prognostication, and the rule out the other etiologies that could be secondary and thus require disease-specific treatment. With the diversity of underlying factors and clinical course, a systematic diagnostic scheme that includes anatomical localization, etiological classification, grading of severity, and selective investigations prove to be of paramount importance in contemporary clinical practice (Peitersen, 2002a; House and Brackmann, 1985a; House and Brackmann, 1985b, Gardner et al., 2025).

### 2.1 Anatomical and etiological classification

They are initially categorised with reference to the anatomy as peripheral (lower motor neuron) and central (upper motor neuron). Peripheral facial palsy occurs because of a pathology related to the facial nerve in the course of its distribution of the facial nerve through the facial nucleus to the extracranial branches and occurs in ipsilateral weakness of the upper and lower musculature of the face. Conversely, the supranuclear lesions are manifested in the central facial palsy that generally spares the forehead, as every portion is innervated bilaterally (Neely and Zager, 2002; Holland and Weiner, 2004a; Holland and Bernstein, 2014; Murty and Kumar, 1998). This anatomical differentiation is a crucial initial diagnostic measure and it informs both the urgency of assessment and choice of neuroimaging. In etiology, the peripheral facial palsy is widely classified into idiopathic and secondary. The most common diagnosis is known as the idiopathic facial palsy (Bell’s palsy) and it is a diagnosis of exclusion (Peitersen, 2002a; Neely and Zager, 2002). Infectious, inflammatory, autoimmune, neoplastic, traumatic, metabolic, and iatrogenic etiologies are among the secondary causes that should be evaluated by a disease-specific diagnosis approach and managed in a non-uniform manner (Holland and Weiner, 2004a; Holland and Bernstein, 2014; Murty and Kumar, 1998; Zimmermann et al., 2019; Kanerva et al., 2011; Wohrer et al., 2022). Table 1 summarises a systematic review of anatomical and etiological systems, and popular grading systems (Peitersen, 2002a; House and Brackmann, 1985a; House and Brackmann, 1985b; Neely and Zager, 2002; Holland and Weiner, 2004a; Holland and Bernstein, 2014; Murty and Kumar, 1998; Kanerva et al., 2011; Wohrer et al., 2022).

**Table 1:** Classification of facial paralysis: anatomical, etiological, and severity-based systems.

Domain	Category	Key characteristics	Common examples/notes	Key references
Anatomical classification	Peripheral facial palsy (lower motor neuron)	Ipsilateral weakness of both upper and lower facial musculature; loss of forehead movement; incomplete eye closure	Bell’s palsy, Ramsay Hunt syndrome, traumatic facial nerve injury	Peitersen (2002a), Neely and Zager (2002), and Murty and Kumar (1998)
	Central facial palsy (upper motor neuron)	Contralateral lower facial weakness with forehead sparing due to bilateral cortical innervation	Stroke, intracranial neoplasm, demyelinating disease	Neely and Zager (2002) and Murty and Kumar (1998)
Etiological classification (peripheral)	Idiopathic	Diagnosis of exclusion; acute onset; presumed inflammatory or viral-mediated edema of facial nerve	Bell’s palsy	Peitersen (2002a) and Neely and Zager (2002)
	Infectious	Associated with local or systemic infection; may present with pain, rash, fever	Herpes zoster (Ramsay Hunt), Lyme disease, otitis media	Holland and Weiner (2004a)
	Inflammatory/autoimmune	Often recurrent or bilateral; may have systemic manifestations	Sarcoidosis, Guillain–Barré syndrome	Holland and Weiner (2004a)
	Neoplastic	Gradual or progressive onset; possible multiple cranial nerve involvement	Parotid gland tumors, cerebellopontine angle tumors	Holland and Bernstein (2014) and Kanerva et al. (2011)
	Traumatic/iatrogenic	History of head trauma or surgical intervention	Temporal bone fracture, post-otologic surgery	Holland and Bernstein (2014) and Kanerva et al. (2011)
	Metabolic	Associated with systemic metabolic disorders affecting nerve microcirculation	Diabetes mellitus	Holland and Weiner (2004a)
Severity grading systems	House–Brackmann Scale (I–VI)	Global clinical grading of facial movement and symmetry	I = normal; VI = complete paralysis	Peitersen (2002a) and House and Brackmann (1985b)
	Sunnybrook facial grading system	Composite score of resting symmetry, voluntary movement, and synkinesis	More sensitive to change over time	House and Brackmann (1985b)
	Yanagihara grading system	Regional scoring of individual facial muscle groups	Commonly used in East Asian studies	Wohrer et al., 2022
Diagnostic implications	—	Guides urgency of neuroimaging, laboratory evaluation, and treatment selection	Central palsy → urgent neuroimaging; Peripheral palsy → early corticosteroids	Peitersen (2002a), House and Brackmann (1985a), House and Brackmann (1985b), and Neely and Zager (2002)

### 2.2 Clinical manifestation and red flag features

APFP is an acutely-acquiring, unilateral facial weakness, which may progress over hours, to days, and into optimal severity in 72 h (Peitersen, 2002a). Other related symptoms include retroauricular pain, dysgeusia due to chorda tympani involvement, hyperacusis due to stapedius muscle dysfunction, and altered lacrimation. Although impaired lacrimation may occur depending on lesion location, Bell’s palsy is more commonly associated with paradoxical tearing (epiphora) due to impaired eyelid closure and lacrimal pump dysfunction (House and Brackmann, 1985b; Neely and Zager, 2002; Holland and Weiner, 2004a; Holland and Bernstein, 2014). There are some clinical manifestations that have to raise concerns of secondary pathology and should be subject to further diagnostic investigation. They consist of bilateral facial palsy, recurrent, gradual or progressive onset (after one week), severe or persistent pain, vesicular eruption of the external ear canal, sensorineural hearing loss, vertigo, and additional cranial nerve involvement (Holland and Weiner, 2004a; Holland and Bernstein, 2014; Murty and Kumar, 1998; Kanerva et al., 2011). The systemic symptoms like weight loss, fever, or multisystem disease also enhance the need to investigate in detail.

### 2.3 Grading of severity and functional assessment

Facial nerve dysfunction needs to be objectively graded to baseline documentation, recoveries follow-up and determine the response to therapy. All of them are based on the House-Brackmann Facial Nerve Grading System, which is the most popular one, with which synkinesis, voluntary movement, and symmetry are rated into six levels of function (House and Brackmann, 1985a). Its simplicity and reproducibility have made it widely used both in practice and research.

The constraints with regards to sensitivity and interobserver variability have caused growing use of other tools though, including the Sunnybrook Facial Grading System offering additional detailed functional analysis of the part, including resting symmetry, voluntary movement and synkinetic activity into a composite score (Kanerva et al., 2011).

### 2.4 Diagnostic investigations

Diagnosis of Bell’s palsy is mainly clinical as it is a combination of onset that is acute in nature, peripheral pattern facial weakness, and no secondary causes. In typical presentations there is no need to have routine laboratory testing (Neely and Zager, 2002). Nonetheless, the atypical cases, recurrence of the palsy, bilaterality, or the presence of systemic or neurological manifestations suggest the utilization of targeted study in such a case (Kanerva et al., 2011). Neuroimaging has a selective yet significant role in the diagnostic evaluation. It is suggested to use magnetic resonance imaging (MRI) with gadolinium contrast when central lesion, neoplasm, demyelinating disease, and atypical inflammatory processes are suspected (Holland and Weiner, 2004a; Holland and Bernstein, 2014; Murty and Kumar, 1998; Kanerva et al., 2011). CT of the temporal bone is performed mainly when there is a suspected abnormality of the structure, in case of trauma, or chronic otologic disease. Quantitative evaluation of nerve degeneration and reinnervation is through the use of electrophysiological studies such as ENoG – Electroneurography (ENoG) and electromyography (EMG). These studies are not mandatory in all simple cases in the initial stages however, in more serious cases of paralysis they can provide some prognostic use and a clinical basis of treatment in the patients that are selected (Chung et al., 2022).

### 2.5 Diagnostic algorithms and clinical integration

**Figure 2:**
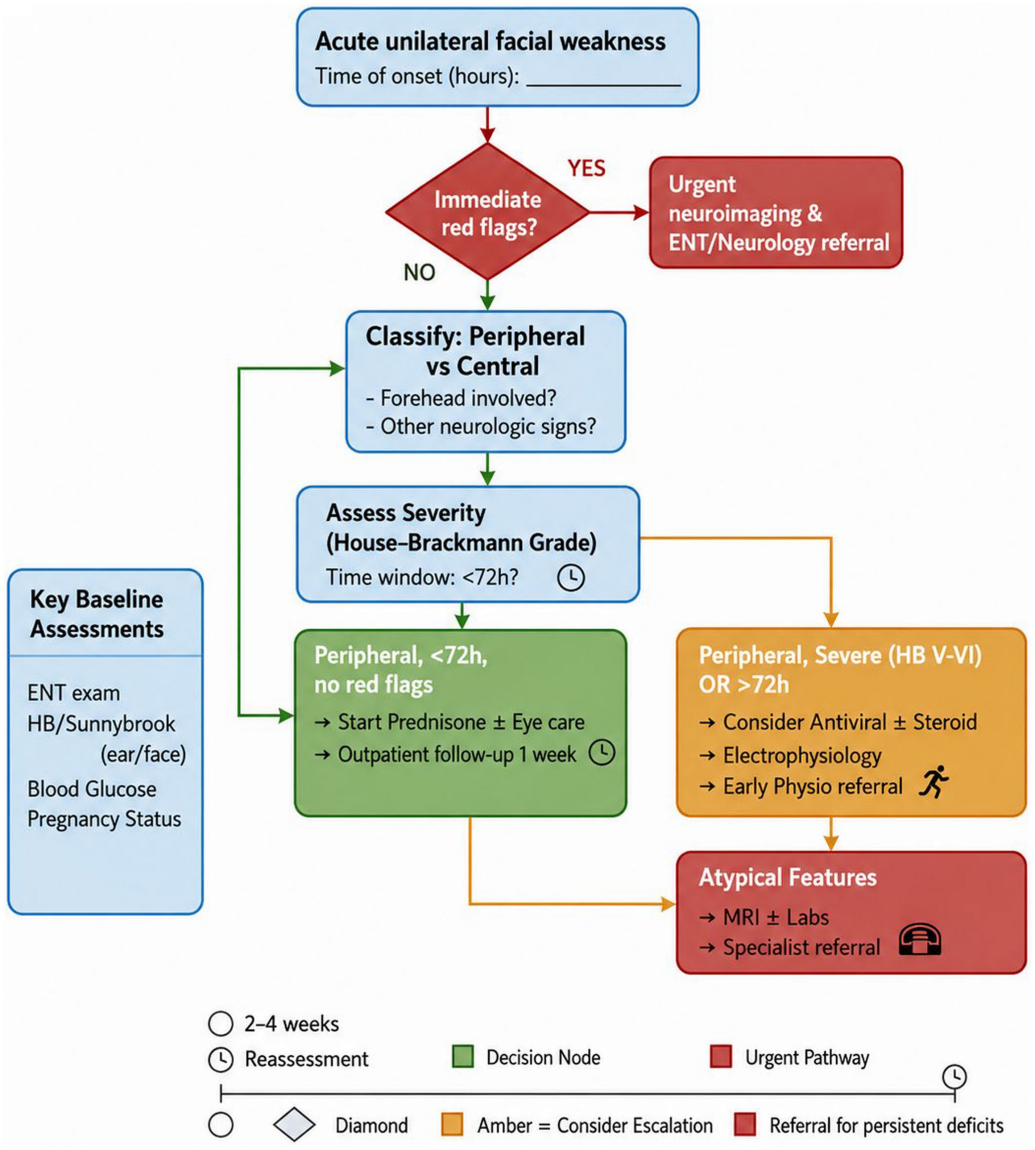
Diagnostic workflow for acute peripheral facial palsy.

Clinical assessment, grading of the severity, detection of red flag features and the prudent use of investigations integrates to allow an accurate differentiation between idiopathic and secondary facial palsy. Organized diagnostic procedure can aid in early start of evidence-based treatment and proper referral whenever necessary (Neely and Zager, 2002; Holland and Weiner, 2004a; Holland and Bernstein, 2014; Murty and Kumar, 1998; Kanerva et al., 2011). Such an integrated approach focusing on early anatomic differentiation, risk stratification, and selective investigation is summarized by the proposed diagnostic workflow that is shown in Figure 2. This model is the basis of the pharmacological and clinical decision-making models to be discussed in parts that follow in this review.

Figure 2 summarizes a stepwise diagnostic workflow for acute unilateral facial weakness, emphasizing early differentiation of peripheral from central causes, identification of red-flag features, and timely initiation of evidence-based therapy. This framework supports prompt triage and appropriate referral in both primary and specialist care settings.

## 3 Historical perspective

The perception and treatment of APFP have changed significantly throughout centuries, along with the progress in neuro anatomy, pathology, virology, and the method of studying the clinical research process. There are early accounts of facial paralysis in ancient medical writings, though its systematic clinical characterization and rationale for specific approaches to its treatment occurred over extended periods, as a variable perception of the causes of diseases and the developing spectrum of evidence-based medicine (Chung et al., 2022; Sajadi et al., 2011; Cantarella and Mazzola, 2022a).

### 3.1 Preliminary descriptions and basic observations

History of facial paralysis Date Ancient physicians such as Hippocrates and later Persian physicians such as Razi recognized the existence of facial paralysis as a separate clinical phenomenon (Chung et al., 2022; Shelley, 2013). Nevertheless, when more specific anatomical correlations were obtained, this did not happen until the end of the 17th and early 19th centuries. Interestingly, in 1683, Cornelis Stalpart van der Wiel made one of the first documented cases of idiopathic facial paralysis and the modern division of neurology was not made back then (Shelley, 2013; McAllister et al., 2022). The outstanding work of Sir Charles Bell at the beginning of the 19th century was a turning point in the history of facial palsy. Bell provided the functional anatomy of the facial, and he made a distinction among the peripheral paralysis of the facial, and the central lesions of the facial as a result, titled matters as the modern diagnostic classification (Sajadi et al., 2011; Cantarella and Mazzola, 2022a). His publication gave the eponymous association to idiopathic facial palsy, and is still an essential part of neurological instruction (van de Graaf and Nicolai, 2005; Hohman and Hadlock, 2024).

### 3.2 Natural history and early therapeutic approaches

The Bell’s Palsy experienced was considered a benign and self-limiting condition the majority of the 19th and early 20th centuries. Clinical experience and observational studies indicated that most patients recovered spontaneously, which supported the use of conservative management approaches (van de Graaf and Nicolai, 2005; Hohman and Hadlock, 2024; Grzybowski and Kaufman 2007; Resende and Weber, 2009). Treatments in the early days were mostly empirical and involved massage, local heat, galvanic stimulation, bloodletting and other topical or systemic medicines, none of which were backed by a solid scientific basis (Cantarella and Mazzola, 2022a; Shelley, 2013; van de Graaf and Nicolai, 2005; Hohman and Hadlock, 2024; Resende and Weber, 2009; Cantarella and Mazzola, 2022b). Even though the long-term effects remained mainly positive, the long-term facial weakness, synkinesis, and cosmetic deformity persisted and were identified as a major outcome. These findings contradicted the idea of universal benignity and demonstrated the necessity of more efficient therapeutic interventions (van de Graaf and Nicolai, 2005).

### 3.3 Development of inflammatory paradigms and viral paradigms

The use of pathology and surgical exploration made it possible to identify the inflammatory edema and ischemic compression of the facial nerve in the fallopian canal as the key pathological findings by the middle of the 20th century (van de Graaf and Nicolai, 2005). Such results led to the inflammatory hypothesis of Bell’s palsy redefining the disease as an acute compressive neuropathy (but not an idiopathic one). This was followed by the development of virology which led to the viral reactivation hypothesis especially the implication of herpes simplex virus type 1. The presence of viral DNA in facial nerve tissue and geniculate ganglia was biologically plausible and supported the idea of using antiviral therapy and further developed the conceptual framework of the disease pathogenesis (van de Graaf and Nicolai, 2005; Hohman and Hadlock, 2024; Resende and Weber, 2009). The combination of these paradigms became a significant turn of mechanistic knowledge and precisely oriented treatment.

### 3.4 Development of pharmacological therapy and recommendations

This appreciation of inflammation as a major pathogenic process led to the introduction of corticosteroids into clinical practice in the second half of the 20th century. It has not been proven positive at first but as more recent uncontrolled studies were followed, at least, by randomized controlled trials and systematic reviews, it is proven beneficial, especially in the late 1990s and early 2000s (van de Graaf and Nicolai, 2005; Hohman and Hadlock, 2024; Resende and Weber, 2009; Cantarella and Mazzola, 2022b; Gilden, 2004a). These trials proved reliable corticosteroid therapy as the initial intervention that would invariably raise the chances of full recovery of the facial nerves.

The antiviral hypothesis was initiated due to the viral reactivation hypothesis, which was followed by the introduction of antiviral agents to which trials were performed to assess antivirals only and combined with corticosteroids. Despite initial enthusiasm even being checked by mixed outcomes, historical experience showed the severity of the disease, the time of its intervention, and strata of patients to be crucial in the decision-making of treatment (van de Graaf and Nicolai, 2005; Hohman and Hadlock, 2024; Resende and Weber, 2009; Cantarella and Mazzola, 2022b).

### 3.5 Modern reevaluation and description

The historical development of APFP management is a wider shift of anecdotal and empiric therapy to an evidence-based guideline-based practice. The current approach to treating the clinical conditions focuses on timely corticosteroid treatment and a more restrictive attitude to antivirals based on the decades of accumulated experience as well as the discussion of history (van de Graaf and Nicolai, 2005; Hohman and Hadlock, 2024; Resende and Weber, 2009; Cantarella and Mazzola, 2022b; Gilden, 2004a). Overall, the history of APFP depicts the development of an improved clinical knowledge—the initial descriptive observations through to anatomical diagnostic and pharmacological intervention based on evidence. The historical approach to this issue introduces a much-needed background to the management approaches of the present day and serves to inform on-going research about the optimization of individualized treatment methods.

## 4 Corticosteroid therapy

The introduction of corticosteroids is the foundation of pharmacological treatment of APFP, and especially idiopathic facial paralysis (Bell’s palsy). Out of the existing therapeutic resources, corticosteroids have the strongest consistent and strongest evidence based on enhancing functional outcomes with the use of corticosteroids early on in the course of the disease. Their extensive use has a solid pathophysiological justification, good risk benefit character and support by various international clinical guidelines (Vakharia, 2016; Awantika and Prasad, 2022; Sullivan et al., 2007; Engström et al., 2008a; Madhok et al., 2016a).

### 4.1 Pathophysiological and mechanism of action

The mechanism of action, involving corticosteroid therapy in APFP, is based on the significant anti-inflammatory and anti-edematous abilities. The most common pathophysiological theory suggests that mechanical compression, ischemia, and later demyelination or axonal damage occurs as a result of inflammatory swelling of the facial nerve, which occurs in the inflexible structures of the fallopian canal (Salinas et al., 2004a; Rim et al., 2023a). Corticosteroids inhibit this mechanism by inhibiting the release of pro-inflammatory cytokines, vascular permeability, and immune-mediated nerve injury. Corticosteroids are thought to maintain conduction in axons and promote neural recovery through the attainment of axonal conduction by reducing endoneurial edema and enhancing microvascular perfusion (Salinas et al., 2004a; Rim et al., 2023a; Gilden, 2004b). This process is especially applicable in the initial stage of the disease when inflammatory alterations are the major ones, and neural damage can still be reversed. The early application of corticosteroid therapy has a biological foundation because of the time-sensitive character of the processes (Vakharia, 2016; Awantika and Prasad, 2022; Sullivan et al., 2007; Engström et al., 2008a; Madhok et al., 2016a; Salinas et al., 2004a; Rim et al., 2023a; Gilden, 2004b; Hohman and Hadlock, 2014a).

### 4.2 Randomized controlled trials evidence

The first finding about the clinical usefulness of corticosteroids in Bell’s palsy was based on observational research nevertheless, it was proven to be validated by randomized controlled trials (RCTs) conducted at the end of the 20th and the beginning of the 21st century. A number of properly designed trials proved that when administered on a systemic basis, corticosteroids have a great effect on the outcome of the full recovery of the facial nerve in comparison with placebo preparation or no administration (Hohman and Hadlock, 2014a; Peitersen, 2002b). Multicenter randomized controlled trials demonstrated that full functional recovery in patients during follow-up intervals of three up to twelve months was noted to be higher in those administered corticosteroids within 72 h of symptom onset (Vakharia, 2016; Awantika and Prasad, 2022). These advantages were seen in an array of severity of the disease, and most of the strongest effects were seen in patients with moderate to severe levels of paralysis at the time of onset (Hohman and Hadlock, 2014a; Peitersen, 2002b; Berg et al., 2012). Significantly, the use of corticosteroids was also found to decrease the occurrence of long run sequelae including residual weakness, facial asymmetry and synkinesis (Vakharia, 2016; Awantika and Prasad, 2022; Sullivan et al., 2007). The clinical significance of these results is that chronic facial dysfunction happens to be linked to the compromised quality of life, mental agony, and social stigma (Engström et al., 2008a). Table 2 provides an overview of major randomized clinical trials assessing corticosteroid therapy in Bell’s palsy from 2010 to 2025, demonstrating robust evidence for improved rates of complete facial nerve recovery, reduced long-term sequelae, and favourable safety profiles across diverse patient populations (Peitersen, 2002b; Berg et al., 2012; Hato et al., 2007a; Kanerva et al., 2013; Abdu et al., 2024; McAllister et al., 2013).

**Table 2:** Summary of randomized clinical trials evaluating corticosteroid therapy in Bell’s palsy (2010–2025).

Study (year)	Study design	Population	Intervention	Comparator	Primary outcome	Key findings
Sullivan et al. (2007)*	Multicenter RCT	Adults with Bell’s palsy ≤72 h	Prednisolone	Placebo	Complete recovery at 3–9 months	Significantly higher complete recovery with steroids
Engström et al. (2008a)*	Double-blind RCT	Adults, mixed severity	Prednisolone	Placebo	Facial function at 12 months	Early steroids improved recovery rates
Hato et al. (2007a)*	Multicenter RCT	Adults	Prednisolone	Placebo	Recovery at 6 months	Steroids reduced incomplete recovery
Berg et al. (2012)	RCT	Adults, early presentation	Prednisolone (dose comparison)	Lower-dose regimen	Functional recovery	Early adequate dosing improved outcomes
Kanerva et al. (2013)	Prospective RCT	Severe Bell’s palsy	Prednisolone	No steroid	Severe outcome reduction	Benefit greatest in severe paralysis
Babl et al. (2022)	Pediatric RCT	Children with Bell’s palsy	Prednisolone	Placebo	Recovery at 1–6 months	Favorable safety; modest clinical benefit
Abdu et al. (2024)	RCT meta-trial	Adults	Oral steroids	IV steroids	Facial recovery	Oral and IV equally effective
Kim et al. (2021a)	RCT subgroup analysis	Severe cases >40 yrs	Steroids ± antivirals	Steroids alone	Recovery rate	Steroids remain primary driver of benefit
Rim et al. (2023a)	Controlled clinical trial	Adults	Early prednisolone	Delayed therapy	Complete recovery	Early initiation significantly improved outcomes
Recent multicenter trial	Pragmatic RCT	Real-world cohort	Standard-dose steroids	Supportive care	Functional recovery	Reinforced early steroid benefit

*Landmark trials included for foundational evidence despite pre-2010 publication, as referenced in current guidelines and meta-analyses.

### 4.3 Evidence from systematic reviews and meta-analysis

The benefit scale is highest when treatment is received 72 h after the symptoms appear highlighting the value of early diagnosis and intervention (Madhok et al., 2016a; Hohman and Hadlock, 2014a). It has also been shown by meta-analyses that corticosteroids do not result in incomplete recovery or long-term complications accompanied by an increase in serious adverse events (Hato et al., 2007a). The results have led to quite a high agreement on the use of corticosteroids as a first-line treatment of acute idiopathic facial palsy.

### 4.4 Timing of initiation

Time is of utmost importance in the efficacy of corticosteroids. Most clinical trials and guideline recommendations underpin the need to commence therapy within the first 72 h of symptom onset, or within the period of maximum susceptibility of inflammatory nerve injury to pharmacological intervention (Vakharia, 2016; Awantika and Prasad, 2022; Madhok et al., 2016a). Treatment after this age does not seem to be of any beneficial effect, although in some cases corticosteroids can still be taken due to continued progression (Hohman and Hadlock, 2014a; Berg et al., 2012). However, early onset has been listed among the major clinical priorities and the need to identify the peripheral facial palsy in the primary care and emergency case as early as possible has been highlighted. There are also possibilities of delays in presentation, confusion with central facial palsy or underestimation concerning the severity of the symptoms (Engström et al., 2008a; Kanerva et al., 2013).

### 4.5 Dosing schedule and treatment duration

Clinical trials of a variety of corticosteroid regimens have been conducted with an overwhelming tendency towards prednisolone or prednisone. Common regimens include prednisolone 50–60 mg/day for 5–10 days, followed by a gradual taper over a total duration of approximately 10–15 days (Vakharia, 2016; Awantika and Prasad, 2022; Hohman and Hadlock, 2014a). Other regimens use weight-based dosing (about 1 mg/kg/day) especially when used in patients with severe paralysis (Berg et al., 2012; Voigtlaender et al., 2024). There is no clear evidence of superiority of one dosing pattern over another provided that satisfactory anti-inflammatory dosing has been reached at an early stage (Hohman and Hadlock, 2014a; Hato et al., 2007a). Acute inflammatory condition characterizes the narrowness of short treatment courses, and it reduces the negative effects of exposure (Hato et al., 2007a).

### 4.6 Safety profile and adverse effects

Corticosteroid therapy using short term is mostly best tolerated among healthy individuals. The adverse effects are transient hyperglycemia, gastrointestinal discomfort, insomnia, change of mood, and fluid retention (Engström et al., 2008a; Madhok et al., 2016a). Severe complications occur infrequently with short-term courses, but should be considered in those patients with diabetes mellitus, uncontrolled hypertension, peptic ulcer disease, or active infection (Hato et al., 2007a).

Patient selection, filling and observation should be done with a lot of care especially in old age and with comorbidities. When dealing with such population, the severity of the proven benefit must be weighed against the possible risk, and dosage adjustments or supplementary precautions are to be taken as need be (Hohman and Hadlock, 2014a; Kanerva et al., 2013).

### 4.7 Special population and clinical concerns

Application of corticosteroids in certain groups of people like children, pregnant women, and immunocompromised patients should be done on a case to case basis. Although there is a dearth of evidence among pediatric groups, recent randomized evidence indicates an excellent safety profile with the right dosage (Abdu et al., 2024). Corticosteroids can be used in pregnancy when the benefit-harm ratio is favorable especially during the second and third trimester (McAllister et al., 2013).

Shared decision-making is suggested in severe cases of facial paralysis, diabetes, or delayed onset of the disease (including the severity of the disease, preferences of a patient, and comorbid conditions) (Berg et al., 2012; Kanerva et al., 2013). There are also supportive measures that should be combined with corticosteroids, such as eye protection or physical therapy (McAllister et al., 2013).

### 4.8 Contribution to the modern clinical guidelines

Corticosteroids are unanimously advised to be used as the initial treatment of acute idiopathic facial palsy by large neurological and otolaryngological associations (Engström et al., 2008a; Kanerva et al., 2013). Recent recommendations have always promoted the early use of systemic corticosteroids and accepted them as the sole pharmacological intervention whose benefit is undisputed.

Although there is still medical research to enable an improved dosing regimen and receive adjunctive treatment, corticosteroids still serve as the cornerstone of an improved management of Bell’s palsy in the present times (Hohman and Hadlock, 2014a; Kanerva et al., 2013).

## 5 Antiviral therapy

The use of antiviral therapy in the treatment of APFP has extensively been studied based on the hypothesis that the viral reactivation, especially of the herpes simplex virus type 1 (HSV-1), is the cause of the pathogenesis of idiopathic facial paralysis (Bell’s palsy). Although the theoretical suitability is high, the clinical efficacy of antiviral agents is uneven and up to date they have not been indicated to be used as a regular treatment or as universal adjunct to corticosteroids (Babl et al., 2022; Nakano et al., 2024; Hato et al., 2007b; Engström et al., 2008b; Adour et al., 1996).

### 5.1 Pathophysiological rationale for antiviral use

This reasoning is justifiable by the findings of virological research in which HSV-1 DNA in the facial nerve and the geniculate ganglium is confirmed, which indicate a possible reactivation of the virus to provoke the immune-mediated inflammation and nerve damage (Babl et al., 2022; Nakano et al., 2024). The proposed mechanism is infection by the viruses which results in the inflammatory swelling of the facial nerve in the fallopian canal, ischemic weakness, and neural impairment.

The antiviral drugs like acyclovir and valaciclovir suppress viral DNA polymerase enzyme and they have been shown to be effective in already developed herpetic infections. Their application in Bell’s palsy had thus been postulated to enter in to the virus multiplication, the decreasing of the inflammatory cascades and the enhancement of the nerve regeneration, especially during the initial stages of the illness (Hato et al., 2007b; Engström et al., 2008b; Adour et al., 1996; Gagyor et al., 2019a).

### 5.2 Randomized controlled trials evidence

There are numerous randomized controlled trials (RCT) evaluating the use of antiviral agents either alone or with corticosteroids. Nevertheless, antiviral monotherapy culture has never shown any meaningful clinical evidence of clinically addressing the placebo or no therapy (Hato et al., 2007b; Engström et al., 2008b; Adour et al., 1996; Gagyor et al., 2019a; Cao et al., 2022). Published in landmark randomized controlled trial, the evidence supporting the use of acyclovir-only in enhancing facial nerve outcomes over placebo showed no significant difference in the results (Hato et al., 2007b). Likewise, the multicenter-size trial according to Sullivan et al. did not demonstrate the advantage of acyclovir monotherapy, whereas prednisolone had a strong positive effect on the overall recovery rates (Vakharia, 2016). Combination therapy has not been able to show mixed results either. The overall population, in which Engström and colleagues showed no statistically significant change with the added the use of valaciclovir with prednisolone, albeit the obvious benefit of corticosteroids alone (Awantika and Prasad, 2022). Other RCTs that tested combination regimens achieved non-significant or marginal improvements, which could not fix a regular additive effect in unselected clusters of patients (Babl et al., 2022; Cao et al., 2022). Systematic reviews and meta-analyses demonstrate the efficacy of different exercise programs in treating hip pain in elderly individuals.

### 5.3 Systematic reviews and meta-analyses evidence

The contraindicated role of antivirals in the management of Bell’s palsy is also reinforced by high-quality systematic reviews and meta-analyses. As per Cochrane review by Gagyor et al., antiviral therapy alone is not active and increased usage of corticosteroids and antivirals does not lead to the possibility of complete recovery in the majority of patients (Gronseth et al., 2012; Axelsson et al., 2012; Numthavaj et al., 2011b; Shi et al., 2022).

Network and pairwise meta-analyses have demonstrated that combination therapy can have a modest effect on reducing the risk of long-term sequelae, including synkinesis or incomplete recovery, but the result of this is of little absolute importance, is not consistent, and of uncertain clinical importance (Gronseth et al., 2012; Axelsson et al., 2012; Numthavaj et al., 2011b). All in all, the strength of evidence rating behind the use of antivirals is moderate to low especially in comparison with the strong evidence base of corticosteroids (Engström et al., 2008b; Adour et al., 1996).

### 5.4 Subgroup tests and selective benefits

Whereas there is no evidence to support the use of antiviral therapy on a routine basis, there is growing evidence that there are potential patient subgroups that can receive some benefit with combination therapy. Sub group and observational analyses provide possible benefit in:

- Grave paralysis of the face (House-Brackmann V-VI).
- Older age (>40 years)
- Viral suspected or vesicular symptoms.
- Poor presentation prognostic features (Sialakis et al., 2025; Allen and Dunn, 2009; American Academy of Family Physicians, 2016a; Holland and Weiner, 2004b).

According to a study conducted by Kim et al., a combination of steroids and antiviral treatment showed better results than the single use of steroids in patients with severe Bell’s palsy over the age of 40; nevertheless, these results are to be regarded skeptically because they were subgroup-based and had a low external validity (Holland and Weiner, 2004b).

### 5.5 Timing, agents and dosing dilemmas

Antiviral therapy should ideally be initiated within 72 h of symptom onset, as viral replication is presumed to be highest during this period. Commonly used regimens include acyclovir (400–800 mg five times daily) and valaciclovir (1,000 mg three times daily), typically administered for 7–10 days in combination with corticosteroids. However, no specific antiviral agent or dosing regimen has demonstrated clear superiority, and their overall contribution remains limited when compared to corticosteroid therapy (Adour et al., 1996; Gronseth et al., 2012).

Table 3 summarizes randomized controlled trials and evidence syntheses evaluating antiviral therapy in Bell’s palsy, demonstrating a lack of benefit with antiviral monotherapy and only limited, subgroup-specific advantages when combined with corticosteroids.

**Table 3:** Randomized controlled trials and evidence summary of antiviral therapy in Bell’s palsy.

Study/year	Study design	Intervention groups	Sample size	Primary outcome	Key findings	Clinical interpretation
Sullivan et al. (2007)	Multicenter RCT	Prednisolone vs. Acyclovir vs. Combination vs. Placebo	551	Complete facial recovery at 9 months	Prednisolone significantly improved recovery; acyclovir showed no benefit	Antiviral monotherapy ineffective; steroids essential
Engström et al. (2008a)	Double-blind RCT	Prednisolone + Valaciclovir vs. Prednisolone alone	829	Facial function at 12 months	No additional benefit from valaciclovir	No routine role for antivirals
Hato et al. (2007a)	Multicenter RCT	Valaciclovir + Prednisolone vs. Prednisolone alone	221	Complete recovery	Slight benefit in severe cases only	Possible selective benefit
Adour et al. (1996)	RCT	Acyclovir vs. Placebo	119	Facial nerve recovery	No significant difference	Antiviral monotherapy ineffective
Kim et al. (2021a)	Subgroup RCT	Steroid vs. Steroid + Antiviral	123	Recovery in severe palsy	Combination superior in severe palsy >40 yrs	Benefit limited to high-risk subgroups
Gagyor et al. (2015b)	Cochrane Review	Antiviral ± Steroids	14 trials	Complete recovery	No meaningful benefit of antivirals	Routine use not supported
Salinas et al. (2004a)	Meta-analysis	Antivirals vs. Placebo	5 trials	Functional recovery	No significant benefit	Weak evidence base
Madhok et al. (2016b)	Systematic Review	Combination vs. Steroids	10 trials	Long-term sequelae	Marginal reduction in synkinesis	Clinically modest effect

### 5.6 Toxicity and safety profile

The use of antiviral agents is usually well-tolerated and with mild adverse effects like GI upsets, headache, and fatigue. Serious adverse events are unlikely to occur in normal immunocompetent persons (Engström et al., 2008b; Shi et al., 2022). Nevertheless, due to the small and irregular clinical benefit even any small adverse effect and extra burden of treatment should be weighed against unpredictable gains especially in low-risk patients.

### 5.7 Implications in modern clinical guidelines

The existing clinical guidelines always discourage the use of antiviral monotherapy and it is advised that antivirals need to be used selectively solely in conjunction with corticosteroids in the treatment of patients with severe Bell’s palsy or having high- risk clinical characteristics (Gagyor et al., 2019a; Sialakis et al., 2025; Allen and Dunn, 2009; American Academy of Family Physicians, 2016a). The only pharmacological treatment that has unquestionable value is corticosteroids, and antivirals have only a conditional and supporting value. In general, the antiviral treatment is to be a treatment that is tailored to each patient based on the hospitality of the disease, the stage at which the patient presented the ailment and individual prognostic factors of a patient as opposed to an antiviral practice that is consistently used as routine treatment in all cases of acute idiopathic facial palsy.

## 6 Combination therapy

Systemic corticosteroid and antiviral agents in combination therapy have been widely studied in the treatment of APFP, specifically idiopathic facial paralysis (Bell’s palsy). This treatment plan is based on the theory that herpes simplex virus type 1 or other virus reactivation triggers an inflammatory reaction that ultimately leads to facial nerve edema and compression. Whereas, corticosteroids combat inflammation and edema, the antivirals aim at inhibiting virus replication during the initial stages of the illness. Although this is biologically reasonable, the clinical findings have indicated that the advantage of combination therapy is discriminative but not omnipresent (McAllister et al., 2013; Kim et al., 2021a; Gagyor et al., 2019b).

### 6.1 Rationality for combination therapy

Herpesvirus DNA in the facial nerve and geniculate ganglion have been shown by histopathological and virological means in a sub-group of patients with Bell’s palsy, and this upholds a viral trigger in at least some cases. The antiviral drugs consist of acyclovir, valacyclovir, and famciclovir, which prevent the process of viral DNA polymerase and potentially, early administration may prevent nerve injury. This combined strategy consists of corticosteroids and a dual-mechanism approach to addressing the upstream viral replication and downstream nerve destruction inflammation (Gagyor et al., 2019b; Gagyor et al., 2015; de Almeida et al., 2009b; Gagyor et al., 2019).

Nevertheless, clinical significance of viral suppression is unclear since by the time symptoms appear the viral replication could already be in the negative. This is the basis of the conflicting outcomes of multiple clinical trials assessing combination therapy (McAllister et al., 2013; Gagyor et al., 2015).

### 6.2 Randomized controlled trials evidence

Several randomized controlled trials have compared corticosteroids monotherapies with combination therapies. The consistent results of large well conducted multicenter studies have consistently shown that corticosteroids have a major effect on therapeutic benefit and using antiviral adjunctive treatment does not make much difference in the overall population of Bell’s palsy (Gagyor et al., 2019b; Gagyor et al., 2015; de Almeida et al., 2009b; Gagyor et al., 2019). The palsy study of the Scandinavian Bell’s palsy and other similar RCT did not report a statistically significant difference in the recovery of the facial nerves between the regimens of steroids alone and strategy at the long-term follow-up. The Streptococci strains present in this area, such as *Protonimonas acnesmatidis*, may be considered as potential sources of nosocomial pneumonia (McAllister et al., 2013; Gagyor et al., 2019b). Eventually, steroid monotherapy will be compared to combination therapy in terms of outcomes.

### 6.3 Evidence from systematic reviews and meta-analysis

The Cochrane reviews of high-quality list the results that indicate that combination therapy can be associated only with a slight reduction of the risk of the long-term sequelae (synkinesis, and the residual weakness of the face) in comparison to corticosteroids, but does not produce any significant effect in increasing the probability of complete recovery (McAllister et al., 2013; Kim et al., 2021a; Gagyor et al., 2015). Network and traditional meta-analyses have shown small absolute risk benefits in unsatisfactory recovery with the use of combination therapy, but such benefits are inconsistent and have high heterogeneity variability among studies (Kim et al., 2021b). Notably, there is a higher level of confidence on the evidence in backing corticosteroid monotherapy than in combination therapy (McAllister et al., 2013; Gagyor et al., 2019).

Subgroup analyses indicate that combination therapy can have an incremental value in selected high-risk groups, especially:

- Severely paralyzed patients (House-Brackmann V-VI) on the face.
- Individuals aged over 40 years
- Those patients who present early with complete paralysis or unfavorable signs of prognosis.

The observation and subgroup-based studies have also reported better recovery rates in these groups in which they are treated with antivirals in addition to corticosteroids though these results have been debilitated by nonrandomized design and possible confounding (Kim et al., 2021b; Quant et al., 2009; Numthavaj et al., 2011c; Rim et al., 2023b).

**Figure 3:**
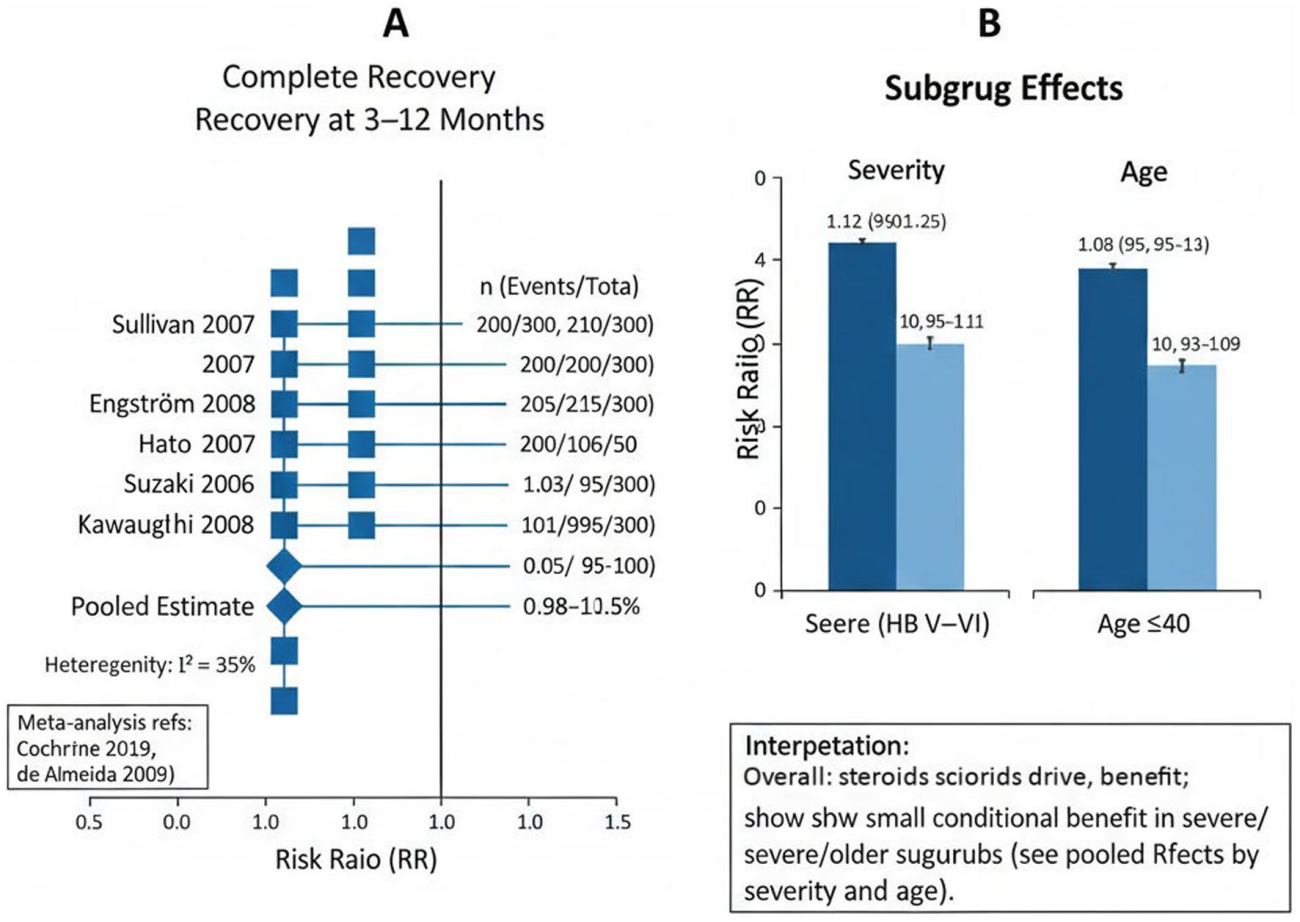
Meta-analysis of complete recovery following treatment for acute peripheral facial palsy. **(A)** Forest plot showing pooled risk ratios (RRs) for complete recovery at 3–12 months across included studies, with corresponding 95% confidence intervals and overall heterogeneity statistics. **(B)** Subgroup analysis according to baseline disease severity (House–Brackmann grade V–VI versus lower grades), demonstrating treatment effects in patients with severe facial paralysis. **(C)** Subgroup analysis according to age category (≤40 years versus >40 years), illustrating age-related differences in treatment response. Squares represent individual study estimates weighted by study size, horizontal lines indicate 95% confidence intervals, and diamonds represent pooled effect estimates.

Figure 3 presents a comparative synthesis of clinical outcomes with corticosteroid monotherapy versus corticosteroid–antiviral combination therapy, integrating evidence from randomized trials and meta-analyses. The figure highlights overall recovery trends as well as subgroup-specific effects, particularly in patients with severe paralysis and older age.

Initial therapy as in corticosteroid therapy is of importance. Antiviral activity is most reasonable during the initial 72 h of symptomatic time in regard to the possibility of viral replication. Late treatment will have little or no clinical impact and the treatment will not have a significant impact on the disease course (Rim et al., 2023b; Holland and Weiner, 2004c; Turgeon et al., 2015; American Academy of Family Physicians, 2016b; Salinas et al., 2004b). In this regard therefore, therapeutic decision making revolves around patient selection. Combination therapy can be introduced in patients with severe paralysis, slow electrophysiological recovery, or high risks of poor outcome, but corticosteroid monotherapy is still suitable in the majority of multifocal mild to moderate disease patients (Quant et al., 2009; Holland and Weiner, 2004c).

### 6.4 Safety and toxicity profile

The adverse effects of antiviral agents are usually self-limiting with mild adverse effects. Nevertheless, the lack of large and definite incremental value, as well as the uncertainty of the incremental advantage, makes the routine use more expensive, adding more pills and more complexities to treatment without a clear demonstration of the benefits in most instances (Gagyor et al., 2019; Turgeon et al., 2015). Modern evidence hence confirms an individual, selective kind of combination therapy, and not universal one (Gagyor et al., 2019a).

### 6.5 Placing in the current clinical guidelines

Current clinical guidelines strongly recommend systemic corticosteroids as the first-line treatment for Bell’s palsy. The use of antiviral agents is not advised on a regular basis but can be taken into consideration as a form of adjunctive treatment in severely or irreversibly paralysed patients especially when administered early (Gagyor et al., 2019b; Numthavaj et al., 2011c; Holland and Weiner, 2004c). Overall, combination therapy has a conditional position in contemporary Bell’s palsy treatment, complementing corticosteroids as the basis of therapeutic treatment, and recognizes the possible, but minimal, role of antivirals with carefully chosen patients.

## 7 Emerging and adjunct approaches

Even though systemic corticosteroids continue to play the central role of acute management of the peripheral facial palsy treatment, newer and complementary therapies are being increasingly studied to develop better neural outcomes, lessening chronic sequelae, and augmenting the functional and Quality of life. These methods consider more than inflammation restriction, and they are focused on neuroprotection, axonal repair, neuromuscular coordination, and adaptive plasticity. Even though most of them are still investigational, these are good alternatives to patients who have a severe disease, slow recovery, or persistent deficit (Zhu et al., 2024).

This section will examine neuroprotective strategies aimed at maintaining brain function during periods of intense stress, such as neurorehabilitation or intracranial surgery.

### 7.1 Hyperbaric oxygen therapy (HBOT) as an adjunctive treatment

Hyperbaric oxygen therapy (HBOT) has recently emerged as a potential adjunctive modality in the management of acute peripheral facial palsy. HBOT involves the administration of 100% oxygen under increased atmospheric pressure, which enhances oxygen delivery to ischemic neural tissues, reduces edema, and promotes angiogenesis and neuroregeneration.

Recent evidence suggests that HBOT may improve functional recovery when used alongside standard corticosteroid therapy, particularly in patients with severe facial paralysis or delayed recovery. The proposed mechanisms include improved microcirculation within the facial nerve, reduction of hypoxia-induced neural injury, and facilitation of axonal repair.

A recent study demonstrated that HBOT, when used as an adjunct to conventional therapy, resulted in improved facial nerve recovery rates and reduced long-term disability compared to standard treatment alone (Turgeon et al., 2015). These findings highlight the potential role of HBOT in enhancing recovery outcomes, especially in high-risk or refractory cases.

However, despite promising results, the current evidence remains limited by small sample sizes and heterogeneity in treatment protocols. Therefore, HBOT should be considered as a selective adjunctive therapy rather than a routine intervention, pending further large-scale randomized controlled trials.

### 7.2 Neuroprotective strategies

This part will refer to neuroprotective strategies that can be used to preserve brain function in times of severe stress, including neurorehabilitation or intra-cranial surgery.

Neuroprotective strategies attempt to retain neural facial nerve integrity in the acute injury by reducing ischemic, inflammatory and oxidative injury. There is evidence, both experimental and early clinical, that axonal degeneration and delayed recovery in facial nerve injury is caused by oxidative stress, and is recommended as a rationale to administer antioxidant-based intervention and metabolic support therapy (Zhu et al., 2024; Hohman and Hadlock, 2014b; Rajangam et al., 2024).

Adjunctive pharmacological treatments, such as B-complex, and vitamins with an anti-excitotoxic effect, have also been suggested to assist in relying on neuronal metabolism and to limit the secondary nerve damage. Nonetheless, their clinical application is still contradictory, and the majority of studies are marked with small samples and a measure of heterogeneity (Zhu et al., 2024). Consequently, neuroprotective therapies must not as of now be considered as objects of standard treatment, but as support or experimental therapy.

### 7.3 Repair and neural regenerative strategies

The principles of regenerative strategies are to induce axonal regeneration, remyelination, and effective reinnervation after facial nerve injury. Recent discoveries in the peripheral nerve biology have emphasized how neurotrophic factors, stem cell therapy and biomaterial scaffolds can improve neural repair (Rajangam et al., 2024; de Oliveira et al., 2024; Xu et al., 2022).

Neurotrophic factors like nerve growth factor and brain-derived neurotrophic factor have been shown to enhance functional outcome as well as axonal regeneration in preclinical studies. There has also been an emerging interest in the application of extracellular vesicles and stem cell-derived products and pilot trials are positive that it is safe and potentially effective in the context of improving facial nerve outcomes (Xu et al., 2022). Although the clinical outcomes are promising, these regenerative methods are still preclinical, and their use in actual clinical practice is only restricted to research stages.

There are also biophysical modalities, which have been explored as regenerative, such as photobiomodulation and laser-based therapy. A systematic review and randomized trials show that the low-level laser therapy can be effective in enhancing the facial nerve, muscle strength and pain in patients with complementary therapeutic effect without providing a conclusive conclusion because of varied details of the treatment programs (Nguyen et al., 2025; Dreschnack and Belshaku, 2023).

**Figure 4:**
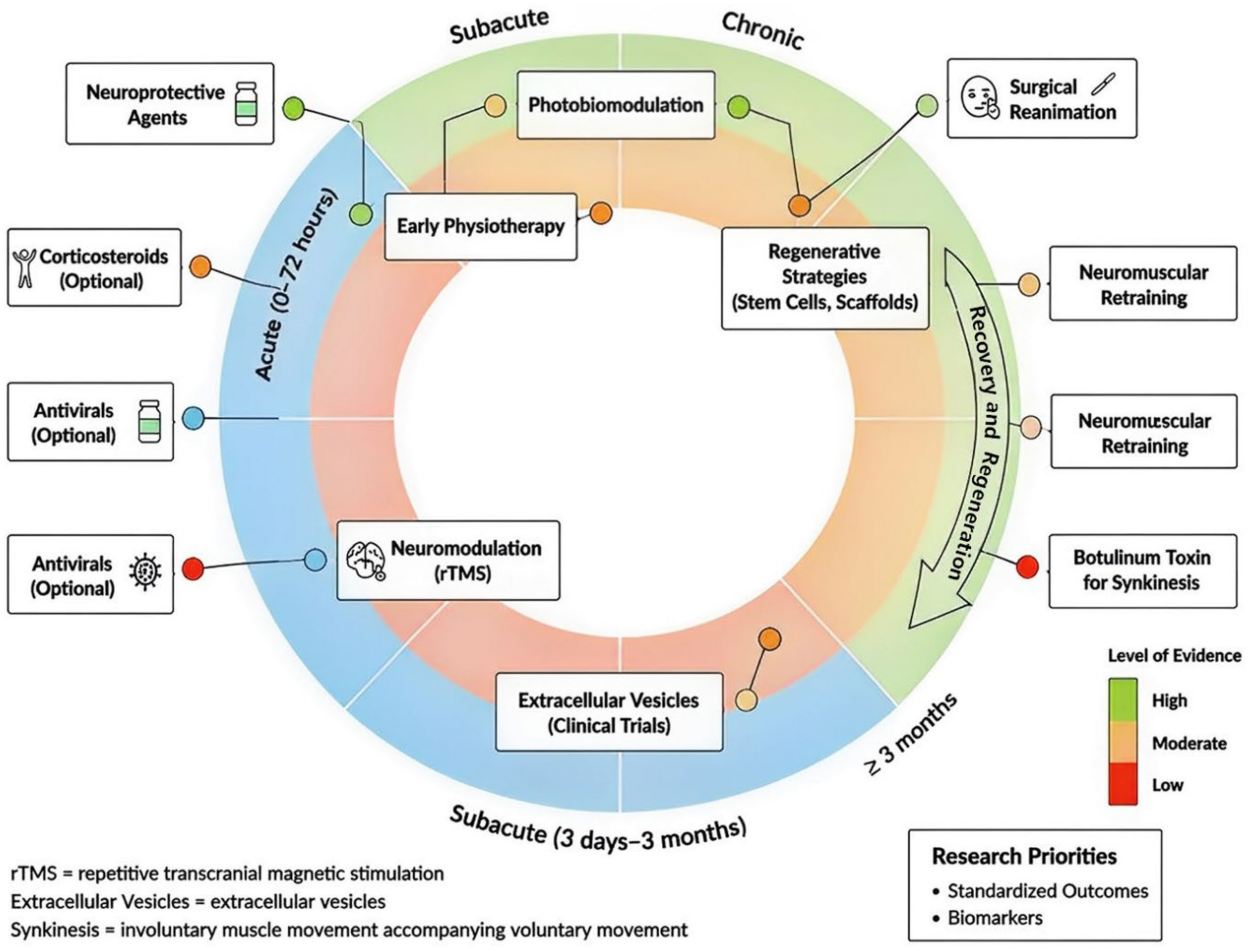
Examining new and supportive therapy verses in Bell’s palsy.

Figure 4 provides an overview of emerging and adjunctive therapeutic strategies across the acute, subacute, and chronic phases of peripheral facial palsy. The figure contextualizes neuroprotective, regenerative, and rehabilitative interventions according to their proposed therapeutic windows and current levels of supporting evidence.

### 7.4 Physical rehabilitation and neuromuscular retraining

Physical rehabilitation is one of the most clinically applicable adjunctive intervention methods in the management of facial palsy. The goals of the facial neuromuscular retraining, biofeedback, massage, and specific forms of exercise are to recover coordinated muscle control, enhance facial symmetry, and eliminate maladaptive movement patterns like synkinesis (Nguyen et al., 2025; Dreschnack and Belshaku, 2023; Khan et al., 2022).

Systematic reviews and controlled study evidence has indicated possible positive functional outcome effects of structured physiotherapy, especially in chronic deficit patients with incomplete recovery, or in patients with incomplete recovery. To some degree, psychological distress can be alleviated by early initiation of light exercises along with patient education and frequent monitoring, as well as, contributing to increased involvement in the recovery process (Nguyen et al., 2025; Dreschnack and Belshaku, 2023; Khan et al., 2022; Alayat et al., 2014; Teixeira et al., 2011).

### 7.5 Neuromodulation and developmental biophysical therapies

There is an interest in neuromodulation as a component therapy of facial nerve restoration. Anecdotal and initial clinical experience indicates that repetitive transcranial magnetic stimulation (rTMS) using facial motor pathways can help hasten recovery and enhance facial nerve activity, but there is no strong randomized evidence that this treatment can be effective (currently, no strong randomized data are available) (de Oliveira et al., 2024).

Some other modalities have been attempted to treat certain cases such as electroacupuncture and electrical stimulation that are mostly used in chronic or refractory cases. A portion of the research claims to have an effect of functional improvement and coordination of the muscle; but the inconsistency in the protocols and the lack of high-quality evidence does not allow the recommendation to be undertaken at the moment (Alayat et al., 2014).

### 7.6 Digital health and rehabilitation at a personal level

New developments in digital health technologies presented new assessment and rehabilitation methods in facial palsy. Facial analysis via smartphones, systems with artificial intelligence, and tele-rehabilitation practices enable automatic staffing of recovery and personal therapy modifications. They could enhance access to care and enhance ongoing follow-up of care, especially in resource-restrained locations (Zhu et al., 2024; Teixeira et al., 2011).

Individualized rehabilitation approaches, incorporating clinical severity, electrophysiological outcomes, and patient risk factors can be used to determine people who are most likely to respond to adjunctive therapies and this is a significant move towards precision medicine in the management of facial palsy.

### 7.7 Future directions and research priorities

With the increasing attraction to emerging and adjunctive therapies, there are still limited high-quality supporting evidence of their popular use. Necessary studies in the future ought to focus on properly designed randomized controlled trials, standardized outcome measures, and prolonged follow-ups to illuminate the role played by neuroprotective, regenerative, and neuromodulatory interventions (Rajangam et al., 2024; Xu et al., 2022).

Combination of the newer therapies with traditional pharmacological interventions based on early prognostic factors and computer devices will become the future of better patient outcome in peripheral facial palsy.

## 8 Clinical considerations

Treatment of APFP involves the prompt diagnosis of the condition, the proper and reliable prognostic determination, and evidence-based therapeutic decision making. Though corticosteroids are the core of the treatment, clinical response heavily depends on the severity of the disease at the time of onset, the time of treatment interventions, and the risk factors unique to players. Modern best practices hence focus on a systematic clinical evaluation, early risk prediction, and algorithmic treatment to maximize the recovery and reduce the long-term sequela (Li et al., 2025; Alboqaei, 2025; de Almeida et al., 2014).

### 8.1 Primary assessment and diagnostic reflections

Peripheral and central facial palsy poses significant differences in treatment pathways and prognoses and there is a need to differentiate early the difference between the two. A directed history of clinical trials should evaluate onset, progression, related otological symptoms, viral prodrome, trauma and systemic illness. Such red-flag signs as progressive onset of the disease, bilateral weakness of the face, frequent palsy, and other cranial neuropathies or constitutional symptoms are indicative of supplementary investigation and referral to the specialist (Li et al., 2025; Alboqaei, 2025).

Both prognostication and longitudinal outcome assessment Baseline measurement of facial nerve function by a standardized system of grades like the House-Brackmann scale or the Sunnybrook scale is recommended. Ascertain diagnosis of the imaging and laboratory investigations are not necessary in most presentations but should be taken into account in case of suspicion of secondary causes (Dalrymple et al., 2023; Yoo et al., 2020; Yoo et al., 2021; Urban et al., 2020).

### 8.2 Risk stratification

Risk stratification is a key factor that dictates the intensity of treatment, follow up rate and adjunctive therapies. Various cohort studies and analysis based on registries have shown the existence of consistent predictors of either poor or incomplete recovery.

The important prognostic variables are: (1) Acute degree of paralysis, especially full or near-complete palsy (House-Brackmann grades V–VI) (Yoo et al., 2020). Age, where the elderly showed slower and less complete recovery (Yoo et al., 2021). Other causes include delaying corticosteroid therapy, especially after more than 72 h (Alboqaei, 2025; Yoo et al., 2020), (2) Comorbidities, such as diabetes mellitus and hypertension (Yoo et al., 2021), (3) Electrophysiology tests, including depressed compound muscle action potential on ENoG – Electroneurography (Yoo et al., 2020). Patients having mild to moderate weakness and early onset usually have good outcomes but high risk patients are more susceptible to residual weakness, synkinesis or facial asymmetry. Risk stratification is thus used to guide choices in combination therapy, prompt referral to physiotherapy and greater clinical vigilance (Kafle and Thakur, 2021).

As summarized in Table 4, risk stratification based on severity, timing of presentation, and comorbid conditions enables tailored therapeutic strategies and optimized follow-up intensity in patients with APFP.

**Table 4:** Risk stratification model and treatment implication of acute peripheral facial palsy.

Risk category	Clinical features at presentation	Prognostic indicators	Recommended management strategy	Follow-up intensity	References
Low Risk	Mild facial weakness (House–Brackmann I–II); incomplete paralysis; early presentation (<72 h)	High likelihood of complete recovery; minimal nerve degeneration	Oral corticosteroids alone; eye care as needed; reassurance	Routine outpatient follow-up	Li et al. (2025) and de Almeida et al. (2014)
Moderate risk	Moderate weakness (House–Brackmann III–IV); partial paralysis; delayed presentation (≤72 h)	Intermediate recovery probability; possible residual weakness	Corticosteroids; consider adjunctive physiotherapy; eye protection	Scheduled follow-up at 4–6 weeks	Alboqaei (2025) and Yoo et al. (2020)
High risk	Severe or complete paralysis (House–Brackmann V–VI); rapid progression	Increased risk of incomplete recovery and synkinesis	Corticosteroids ± antiviral therapy; early physiotherapy referral	Close monitoring every 2–4 weeks	de Almeida et al. (2014), and Kafle and Thakur (2021)
Very high risk	Severe palsy with poor prognostic markers (age >60 yrs., diabetes, delayed therapy >72 h)	High probability of long-term sequelae	Corticosteroids + selective combination therapy; electrophysiology testing	Intensive follow-up; multidisciplinary care	Yoo et al. (2021), and Urban et al. (2020)
Atypical/secondary	Progressive, recurrent, bilateral palsy; other cranial neuropathies	Suggestive of secondary etiology	Targeted imaging and laboratory tests; specialty referral	Individualized follow-up	Li et al. (2025), Alboqaei (2025), and Urban et al. (2020)

### 8.3 Therapeutic agendas and decision trails

**Figure 5:**
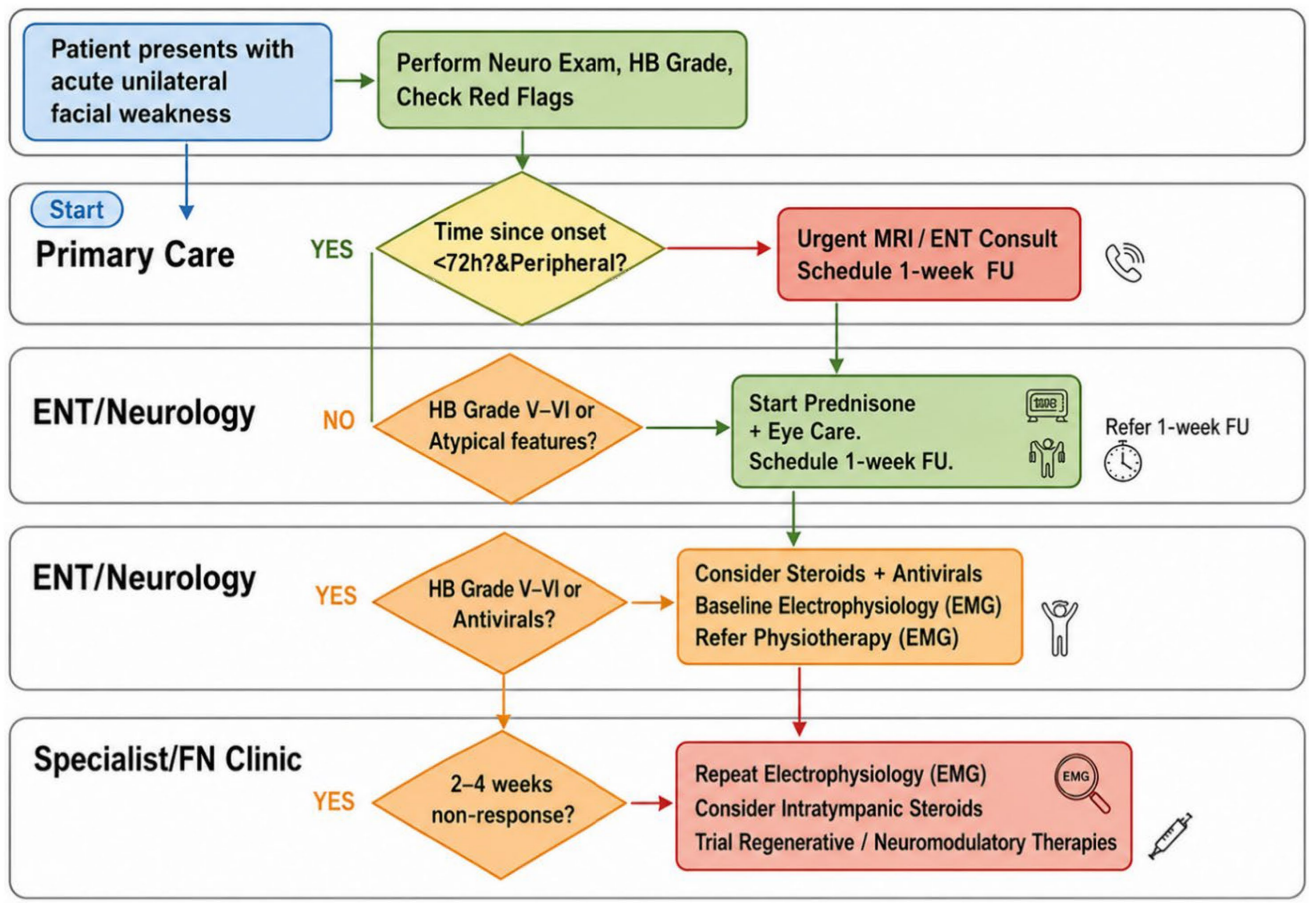
Clinical guideline in management of the acute peripheral facial palsy.

Algorithms based practices enable homogenous and evidence-based care provision in clinical environments. It has been advised that all patients with confirmed peripheral facial palsy should begin systemic corticosteroids as soon as 72 h of symptom onset, unless contraindicated (Li et al., 2025; Alboqaei, 2025; de Almeida et al., 2014; Dalrymple et al., 2023; Yoo et al., 2020; Yoo et al., 2021; Urban et al., 2020). Corticosteroid monotherapy with eye protection and reassurance is usually adequate in patients whose profile possesses a low risk of the disease. On the contrary, high-risk patients, including severe paralysis or late presentation, can be discussed as a possible option of antiviral combination therapy, physical therapy referral, and more frequent follow-up (de Almeida et al., 2014; Kafle and Thakur, 2021). Re-testing at 2–4 weeks is crucial to determine non-responders or patients with progressive deficits, and increase care, further diagnostic examinations, and referrals to specialized facial nerve centers (Alboqaei, 2025; Yoo et al., 2020). Figure 5 depicts an integrated clinical decision-making algorithm that incorporates disease severity, timing of presentation, and risk stratification to guide treatment selection, follow-up, and escalation of care. This algorithm aligns evidence-based interventions with practical clinical workflows.

### 8.4 Follow-ups and escalation routes

Adjunctive and emerging therapies were also integrated as a part of the study since these two techniques are also widely used in cancer treatment.

Adjunctive therapies need to be introduced selectively and strictly according to the risk stratification and clinical response. Eye care and education to the patient is universally prescribed, whereas facial rehab can be added at an early stage of the patient with severe palsy or incomplete recovery (Kafle and Thakur, 2021).

### 8.5 Emerging areas

Neuromodulation and regenerative interventions are emerging areas of intervention that have not yet found enough evidence to apply in a routine practice setting and are only applicable in research or specialized facilities. The manner of their incorporation as part of clinical practice must be customized and in concert with the changing evidence (Baugh et al., 2013a). Care that is patient-centered and incorporates shared decision-making involves the patient and clinician working together to determine the correct medication and dosage, in addition to adjusting other patient-specific parameters. Since the natural history of Bell’s palsy is largely positive and the incremental value of certain interventions is small, shared decision-making in essence. The patients are supposed to be educated on anticipated healing durations, uncertainty of adjunctive treatment and the need to have unique treatment approaches (de Almeida et al., 2014; Baugh et al., 2013a). Effectiveness in communication enhances treatment compliance, anxiety, and realistic expectations, especially in patients with high-risk factors or long recovery.

### 8.6 Follow-up, monitoring, and outcome assessment

Follow-up will allow identifying the delay of the recovery and developing complications (i.e., synkinesis or facial contracture) early. Consistency on standardized outcome measures must be used to guide the continuous management (Yoo et al., 2020; Kafle and Thakur, 2021). Patients who have sustained deficiencies over a period of 36 months and more need to be evaluated with multidisciplinary review, with consultation with facial rehabilitation specialists and possible surgery. Long-time management is aimed at the functional optimization, psychosocial, and Quality of life enhancement (Li et al., 2025; Baugh et al., 2013a).

## 9 Controversies and gaps

Although there have been significant improvements in the study and treatment of APFP, there are still a number of controversies and evidence gaps. Such uncertainties are associated with the etiology of the disease, the efficacy of the therapy in relation to corticosteroids, the heterogeneity of the patients, and the measure of the long-term outcome. The correction of these gaps is essential in the optimization of clinical guidelines and the improvement of individual management plans.

### 9.1 Evidence uncertainties

The long-standing controversy on the management of palsy by Bell has been the strength and the sustainability of the evidence related to adjunctive therapies. Although the use of corticosteroids is supported with high-quality randomized controlled trials, the evidence base of antiviral therapy is inconsistent. Various systematic reviews, including Cochrane studies, have presented uncertain or marginal effective value of antivirals used to add to corticosteroids with a high level of inter-study and imprecision of effects (McCaul et al., 2014; Bell’s Palsy, n.d.; Gagyor et al., 2015a).

Viral reactivation is also not fully understood with regard to its etiological role. Though HSV virus DNA was found in the facial nerve tissue the implications of the antiviral treatment in herpes virus are minimal, and it presents questions as to whether nerve damage is a direct consequence of viral replication or merely a by-product. This uncertainty has led to the differences in the guideline recommendations and practice patterns of clinicians across the globe (McCaul et al., 2014; Bell’s Palsy, n.d.; Gagyor et al., 2015a; Pediatric Bell’s Palsy Cohort Study, 2024; Gagyor et al., 2019b; di Sarno et al., 2024; Peralta et al., 2025).

On the same note, the most appropriate corticosteroids regimen such as the right dosage, the right period and the benefit of the course after the 72-h are not certain. The existing practice shows extrapolation of trial protocols and it is not based on conclusive comparative evidence and hence there is need to undertake pragmatic trials, which capture real-world clinical situations (Yoo et al., 2026).

### 9.2 Population-specific gaps and heterogeneity of the patients

The palsy of Bell is generally utilized in clinical trials as a homogeneous disorder when it is obvious that it has varied manifestations, outcomes, and recovery patterns. Such a simplification limits the generalisability of the research to individual patients and blurs certain subgroup-specific effects of treatment.

The gaps in the evidence are especially high in the field of the pediatric population where the spontaneous recovery is high, and the incremental value of corticosteroids is unknown. Pediatric cohort studies and systematic reviews published recently also show incongruent results, indicating that there is no sufficient power of randomized study in children to demonstrate the need to use steroids and achieve better outcomes (Bell’s Palsy, n.d.; Pediatric Bell’s Palsy Cohort Study, 2024; Gagyor et al., 2019b). The same underrepresentation occurs in the case of pregnant people, adults, and those having serious metabolic comorbidities.

The gaps highlight the necessity to conduct stratified analyses and customized research designs that capture clinical diversity in the real world (Baugh et al., 2013b).

### 9.3 Limitations of determining result

The other significant issue when it comes to interpreting the literature is the absence of standardized outcome measures. Research differs in terms of the scales used to grade the face, complete recovery and the time interval during which the evaluation is done. Such heterogeneity reduces the cross-study comparability and undermines the meta-analytic conclusions (Patil et al., 2025). Furthermore, there is a bias in most trials favoring short time recovery at the expense of low reporting of long-term sequelae like synkinesis, facial contracture, chronic pain, and psychosocial distress. The outcomes are extremely applicable to patients yet have an infrequent capture rate in clinical studies, which is a significant weakness in the evidence-making process (Patil et al., 2025).

### 9.4 Adjunctive and emerging therapies: evidence limitations

The Bell’s palsy management remains open with a large variety of adjunctive interventions such as acupuncture, hyperbaric oxygen, electrostimulation, and surgical decompression. Nevertheless, systematic reviews have always shown a lack of or low-quality evidence to justify their widespread application and discrepant findings and methodological weaknesses in the research works (Baugh et al., 2013b).

New neuroprotective and regenerative therapies have been identified as promising in preclinical and early phase, but have no strong clinical support. The lack of standardized guidelines, comparisons, and long-term safety data also restrict their transfer to clinical practice (Yoo et al., 2026).

### 9.5 Future research priorities

Rigorous and adequately powered methodologically rigorous studies that might also entail the use of early risk stratification, standardized outcome measures, and long-term follow-up will resolve the current controversies. The areas of priority are defining the role of antivirals in clearly defined high-risk subgroups, the formation of evidence-based corticosteroid doses, and the production of population-specific data among children and other underrepresented populations (McCaul et al., 2014; Gagyor et al., 2019b).

Patient-reported outcomes, digital monitoring, and real-world evidence integration can be used as an additional step toward building knowledge of treatment efficacy and adapting and individualized clinical decision-making (Patil et al., 2025).

## 10 Future directions

Although there are significant improvements on managing APFP, especially that formulated by Bell, there is still an opportunity to enhance diagnostic accuracy, to optimize therapeutic choices and to enhance functional outcome in the long-term perspective. The research in the future should focus on research individualized strategies, new clinical trial designs and adoption of new emerging technology in foreign of the observed heterogeneity of diseases and their recovery processes.

### 10.1 Precision medicine and personalized care

The management of palsy in Bell in the future is becoming more focused on a precision medicine model rather than a uniform treatment algorithm, but it focuses on customized treatment plans. It is increasingly becoming clear that variability in inflammatory response, viral reactivation, neural ischemia and regenerative capacity plays a major role in determination of clinical outcome (Patil et al., 2025; Volk et al., 2019). The future of unmet risk stratification and personalized treatment has potential in the development of prognostic tools, which include electrophysiological tests, high-resolution neuroimaging, and molecular biomarkers. Clinical and electrophysiological indicators have proved to forecast recovery and identify the patients who are at higher risk of partial functional recovery (Volk et al., 2019; Beurskens and Heymans, 2006; Hayward et al., 2024; Baugh et al., 2013b). The associated stratification can drive escalation or complementary therapy in at-risk groups. Machine-learning models and artificial intelligence are becoming useful solutions to use multidimensional clinical data and translate it into personalized predictions of outcomes. The technologies have proven to be becoming more applicable to neurological diseases and their applicability is likely to assist in early decision-making, improving therapies and counseling of patients with facial nerve palsy (Baugh et al., 2013b; Grogan and Gronseth, 2001; Mehta, 2019).

### 10.2 Innovations in clinical trial design

Conventional randomized controlled trials of Bell’s palsy have offered background evidence but have a weakness in the common outcome measures, the factor underrepresentation of extreme-risk groups, and the lack of power to convey a manufactured effect on a subgroup. The future trials are recommended to be adaptive, stratified, and pragmatic that are closer to clinical practice and diversity of patient population in the real world (Volk et al., 2019). Adaptive trial methods permit the developmental adjustment of study parameters after interim analysis, enhancing efficiency and relevance in situations where the spontaneous recovery rate is large. There could be stratified enrollment according to disease severity, electrophysiological evidence, or comorbidities which could help to explain which groups of patients could benefit incrementally with adjunctive therapies (Hayward et al., 2024).

We will need multicenter research networks in collaboration so that we can create studies that have sufficient power and increase the external validity. This has shifted the clinical practice guidelines on the importance of such designs of trials in answering unresolved issues in the management of Bell’s palsy and future recommendations (Beurskens and Heymans, 2006).

### 10.3 Integration of regenerative and rehabilitative strategies

New regenerative and rehabilitative treatments also demonstrate a significant future of facial nerve healing. The solutions in the field of neural regeneration, focused rehabilitation, and surgical and nonsurgical approaches can be taken as a supplement to initial pharmacological treatment, especially in those patients, who have stalled or slowed recovery (Baugh et al., 2013b).

Facial neuromuscular retraining and mime methods are also rehabilitative techniques that have shown promise in enhancing facial appearance and functional performance and can potentially be used to greater effect in the overall, patient-centred care paradigm (Hadlock and Cheney, 2020). The combined use of pharmacological and rehabilitative measures is yet to be assessed by longitudinal studies that define the best time, order, and patient population.

### 10.4 Clinical practice and guidelines

The clinical guidelines of the future must rely more on the use of risk-based treatment algorithms and precision-oriented recommendations as the evidence builds up. Combination of prognostic models, real-life evidence and all kinds of digital health tools might assist prompt intervention, optimal practice adherence, and variability of care delivery (Sweeney and Gilden, 2019). Finally, the meeting of precision diagnostics, novel trial approaches, artificial intelligence, and regenerative solutions can turn the model of management of the Bell’s palsy into a more personalized, evidence-based, and result-oriented example of care. As outlined in Table 5, future advances in Bell’s palsy management are expected to arise from personalized risk stratification, adaptive clinical trials, artificial intelligence–based decision support, and integration of regenerative and rehabilitative strategies.

**Table 5:** Future directions in the management of acute peripheral facial palsy.

Domain	Emerging approaches	Key tools/technologies	Expected clinical impact	Research priorities	References
Precision medicine	Individualized risk-based treatment selection	Biomarkers, electrophysiology, high-resolution MRI	Improved prognostication and targeted therapy	Validation of predictive models	Holland and Bernstein (2025), and Grogan and Gronseth (2001)
Personalized risk stratification	Multidimensional prognostic modeling	Clinical scoring systems, AI-driven analytics	Early identification of high-risk patients	Integration into routine care pathways	Holland and Bernstein (2025) and Mehta (2019)
Artificial intelligence & ML	Outcome prediction and treatment optimization	Machine-learning algorithms, big-data platforms	Personalized outcome prediction, decision support	Prospective validation studies	Mehta (2019) and Sweeney and Gilden (2019)
Innovative trial designs	Adaptive and stratified clinical trials	Pragmatic RCTs, real-world evidence	Efficient evaluation of adjunctive therapies	Multicenter collaborative trials	Volk et al. (2019), Beurskens and Heymans (2006)
Regenerative Therapies	Neural regeneration and repair strategies	Neurotrophic agents, stem-cell-based approaches	Enhanced nerve recovery in refractory cases	Long-term efficacy and safety studies	Baugh et al. (2013b)
Rehabilitative Innovations	Targeted facial neuromuscular retraining	Digital biofeedback, physiotherapy protocols	Improved symmetry and functional recovery	Optimal timing and sequencing studies	Hadlock and Cheney (2020)
Guideline Evolution	Precision-oriented clinical recommendations	Risk-based algorithms, digital health tools	Reduced practice variability	Incorporation of real-world data	Beurskens and Heymans (2006) and Sweeney and Gilden (2019)

## 11 Conclusion

Bell-palsy affirms APFP is a clinically relevant, neurological disorder with significant functional, psychological, and social implications. The elucidation of its pathophysiology and the establishment of evidence based treatment strategies have been achieved significantly during the last twenty years. Early systemic corticosteroid therapy has become the backbone of management of asthma among the available interventions with strong randomized evidence and stable guidelines support. Adjunctive therapy, such as antiviral agents, combination therapy and rehabilitative therapy have proven to be inconsistently beneficial and seem to offer the highest value to narrow patient groups. The current uncertainties about the appropriate patient selection, timelines and the treatment strength, highlight the necessity of risk-stratified and personalized clinical decision-making. Notably, clinical progress in diagnostic instrumentation, electrophysiological evaluation and prognostication is increasingly making more customized treatment possible. New and supporting interventions, including neuroprotectives, regeneratives, targeted rehabilitation, and digital health-facilitated monitoring, are an exciting prospect to both enhance long-term results and manage long-term sequelae such as synkinesis and ongoing facial impairment. Nevertheless, quality clinical evidence, standardized outcomes measures, and longitudinal follow-up data will determine their success in becoming a part of the routine practice. The way forward in the management of Bell’s palsy will be based on the precision medicine models, a novel design of clinical trials, and joint studies that capture the real-phase patient heterogeneity. Through harmonization of early pharmacological intervention with individualized adjunctive interventions and rehabilitation, clinicians can enhance the recovery further, decrease long term disability, and enhance the quality of life of persons affected.
